# Sensory Processing Issues and Their Association with Social Difficulties in Children with Autism Spectrum Disorders

**DOI:** 10.3390/jcm8101508

**Published:** 2019-09-20

**Authors:** Nada Kojovic, Lylia Ben Hadid, Martina Franchini, Marie Schaer

**Affiliations:** 1 University of Geneva, 1211 Geneva, Switzerlandmarie.schaer@unige.ch (M.S.); 2 Fondation Pôle Autisme, 1204 Geneva, Switzerland; martina.franchini@pole-autisme.ch

**Keywords:** autism, sensory processing issues, adaptive behavior, eye-tracking, social cognition, social difficulties

## Abstract

Sensory processing issues have been frequently reported in individuals with Autism Spectrum Disorders (ASD), but their relationship with social and overall adaptive functioning has not been extensively characterized to date. Here, we investigate how sensory processing atypicalities relate with deficits in social skills, impaired social cognition, and general adaptive functioning in a group of preschoolers with ASD. Sixty-four children with ASD aged 3 to 6 were included in this study, along with 36 age-matched typically-developing (TD) peers. Parent-reported measures of sensory processing, social difficulties and overall adaptive functioning were collected for all children. We also obtained precise measures of social attention deployment using a custom-design eye-tracking task depicting naturalistic social scenes. Within the group of children with ASD, higher intensities of sensory issues were associated with more prominent social difficulties and lower adaptive functioning. We also found that children with ASD who had more sensory issues showed visual exploration patterns of social scenes that strongly deviated from the one seen in the TD group. The association of sensory processing atypicalities with “higher-order” functional domains such as social and adaptive functioning in children with ASD stresses the importance of further research on sensory symptoms in autism.

## 1. Introduction

Autism Spectrum Disorders (ASD) are a group of pervasive neurodevelopmental disorders characterized by difficulties in social communication and interaction, as well as patterns of repetitive behaviors and restricted interests [1]. Autism symptoms can have widespread implications on the academic and professional achievements of the affected individuals, on their everyday functioning, and their integration in society. In young children, early behavioral symptoms pathognomonic for the diagnosis are mostly social, including for instance atypical eye contact, delayed development of the non-verbal and verbal communication skills, and generally diminished interest in social interaction. It is now widely recognized that early and intensive intervention targeting the development of social skills can dramatically reduce the severity of the autism symptoms in the long term and improve adaptive functioning of affected individuals [2,3,4]. There is, however, a dearth of knowledge about the factors leading to reduced social development in children with autism and on the relationship between social and non-social symptoms in autism. A better understanding of the mechanisms leading to the emergence of autism symptoms is, however, critical for improving existing therapeutic strategies and alleviating the impact of the symptoms on the person’s adaptive functioning.

Among the potential candidates that can help better understand the emergence of autism symptoms, altered sensory processing has only recently received increased attention [5,6]. With the DSM-5 [1], sensory processing issues have been included as one of the primary features among diagnostic criteria for the disorder. However, sensory particularities have been reported since the first descriptions of autism [7] and encompass a broad range of difficulties. For instance, individuals with ASD might be oblivious to sensory input (hypo-responsiveness) [8], show exaggerated response to sensory stimuli (hyper-responsiveness) [9], or take pleasure from sensory activities and search for sensory stimulation by engaging in activities such as fidgeting or repeating noises (sensory seeking) [10,11,12,13]. Unusual sensory behaviors (for reviews see Refs. [14,15]) are frequently reported in ASD, with a prevalence ranging from 45 to 95% depending on factors such as age, IQ and the type of control group used [11]. Sensory atypicalities are persistent over the life span and across different levels of cognitive functioning [16] and have important repercussions on adaptive functioning of individuals with ASD [17]. We, however, need to better understand the relative contribution of the “basic” sensory processes to the emergence of “higher-order” autistic features, such as social impairment or patterns of restricted interests.

Sensory processing is crucial to forming reliable percepts of the environment, and disturbances in this process can have broad implications for many areas of functioning of an individual [18]. Sensory processing difficulties strongly correlate with levels of autistic traits in the general population [19,20], suggesting potential contribution of sensory atypicalities to social difficulties in ASD. Moreover, recent findings from prospective studies support the idea that sensory difficulties might even precede difficulties in social functioning and that the two of them might be more closely related than considered initially [5,12]. Children at familial risk of developing ASD (Sibs ASD) showed higher levels of sensory seeking behaviors at 18 months and these were predictive of the later social difficulties measured by Autism Diagnosis Observation Schedule, 2nd edition (ADOS-2) at 36 months [12]. Moreover, the same study shows that the relation between sensory seeking and subsequent social difficulties was mediated by reduced social orienting in the group of Sibs ASD. Thus, the sensory-seeking patterns influenced future social difficulties in Sibs ASD through the association with reduced social orienting. A replication study with a community sample of children at risk for developing ASD [5] corroborated findings of the relation between sensory seeking patterns in the latter half of the second year and social difficulties between ages 3 to 5 years old. This relation was once again shown to be mediated by reduced social orienting [5].

Sensory issues might indeed be central to autistic symptomatology and even precede and predict the emergence of social difficulties and repetitive behaviors in the developmental cascade [5,12,21]. The first signs of sensory aberrance can encompass lower sensorimotor abilities, enhanced visual search, and attention to detail. They appear before the emergence of evident behavioral signs of autism and predict future diagnosis [21,22,23]. Both behavioral and neurophysiological findings suggest that children with ASD show difficulties in suppressing targets surrounding visually-attended area, which explains why enhanced visual search is often reported in this population [24]. However, this atypical spatial attention profile can dramatically affect the way the relevant information is distinguished from the irrelevant one. Furthermore, difficulties in the integration of information coming from different sensory modalities have been described in ASD, with primary cerebellar disturbances being suggested as critical in the developmental cascade resulting in multisensory processing anomalies in this condition [25]. It is possible that from the earliest stages of the development, the difficulty to integrate the elements of the dynamic social information into a stable representation might render social information less appealing and noisy [21]. For instance, it is thought that disturbances in early sensory processing could lead to the atypical deployment of attention to social stimuli, translated as diminished attention toward the eyes and increased attention to mouth and the body [26,27]. Eyes convey information that is subtle in nature, while the information conveyed through mouth and body might be more accentuated and salient and potentially more easily integrated into a reliable stable concept. The exact nature of mechanisms through which sensory processing atypicalities might impact the genesis and/or maintenance of “higher-order” difficulties is largely unknown. A better understanding of the relationship between sensory processing and social functioning might help to improve therapeutic interventions.

Here, we aim at further understanding the association between sensory processing difficulties/atypicalities, and social deficits and overall adaptive functioning in young children with ASD. For that purpose, we used a variety of standardized clinical measures in a sample of 64 preschoolers with ASD and an age-matched control group (age range: 3.0–5.9). We hypothesized that the presence of sensory difficulties would be related with more pronounced social difficulties, corroborating results from previous studies [6,28]. To precisely delineate atypicalities in the deployment of social attention in children with ASD, we administered a passive viewing eye–tracking task consisting of naturalistic social scenes and developed custom tools to quantify the extent to which each child with ASD diverged in the way he/she was exploring the social scene as compared to the group of typically-developing (TD) children. Finally, we were interested in the impact of sensory and social on adaptive functioning. In line with previous studies [29,30,31], we hypothesized that more pronounced sensory issues would be related to significant difficulties in everyday functioning in the group of patients.

## 2. Material & Methods

### 2.1. Participants

The participants in the current study were recruited from the Geneva Autism Cohort [32,33], a larger project aiming at measuring trajectories of development in children with ASD. The University’s Institutional Review Board, under the ethical approval code Psy 12–163, approved this study. All families provided written informed consent to participate.

Here, we included a subsample of the Geneva Autism Cohort, namely a cross-sectional sample constituted from children older than 3 years old for whom we obtained parents-completed Short Sensory Profile (SSP; [34]) and had sufficiently good eye-tracking data for the Social scenes paradigm (see *2.3*). Prior to inclusion, all children with ASD received a clinical diagnosis according to the DSM-5 criteria [1] and the clinical diagnosis was further corroborated using gold-standard diagnostic assessments (see *2.2*). TD preschoolers were initially screened for the presence of any known neurological or psychiatric illness in any first-degree relative and the child itself. Standard diagnostic assessments were also used with all TD children to exclude the presence of autism symptoms. Our final sample was composed of 64 children with ASD (9 females, aged 3.0 ± 0.7 years old, range: 3.0–5.9) and 36 TD children (10 females, aged 3.9 ± 0.7 years old, range: 3.1–5.8).

### 2.2. Clinical Assessments

For all children, a direct measure of autism symptoms was obtained using the Autism Diagnostic Observation Schedule (ADOS-G & ADOS-2; [35,36]), a semi-structured, standardized assessment of communication, social interaction, and restricted interests and repetitive behaviors. Following the ADOS guidelines, we used different ADOS modules according to the children’s age and language level. The ADOS Calibrated Severity Score (ADOS-CSS; [37]) was used as a standardized measure of autism symptoms severity across modules. Calibrated Severity Scores were also obtained by the domain of core symptoms, i.e. for Social Affect (SA-SS) and Restricted interests and Repetitive Behaviors (RRB-SS) [38,39].

We used the Short Sensory Profile (SSP; [34]) to assess sensory processing. The SSP is a 38-items parents-reported questionnaire developed from the long version of the Sensory Profile (SP; [40]) and validated for children aged 3 to 10 years old. The SSP measures sensory responses to environmental stimuli across different modalities: (1) Tactile Sensitivity (TS), (2) Taste/Smell Sensitivity (T-SS), (3) Movement Sensitivity (MS), (4) Underresponsiveness/Sensation-Seeking (U-SS), (5) Auditory Filtering (AF), (6) Low Energy/Weak (L-WE), and (7) Visual/Auditory Sensitivity (V-AS). Parents rated each behavior on a 5-point Likert scale to assess its frequency (1 = frequently occurring, 5 = rarely occurring). For each modality, we obtain a score, and the 7 scores are then summed up to get a Total Score. In the SSP, lower scores indicate higher differences in sensory processing.

In addition to the ADOS, we also collected a parent-reported measure of social difficulties for all children, using the Social Responsiveness Scale, second edition (SRS-2 [41]). The SRS-2 is 65-items scale measuring deficits in social behaviors highly associated with ASD and is widely used as a screener for the diagnosis of ASD. It is composed of different subscales: (1) Social Awareness, (2) Social Cognition, (3) Social Communication, (4) Social Motivation, and (5) Restricted interests and Repetitive Behaviors. All responses are then summed up to obtain a Total score. Additionally, two subscales specific to the DSM-5 diagnosis criteria for ASD are also available (Social Communication Index-SCI, Restricted interests and Repetitive Behaviors Index-RRBI). For each statement, parents were asked to rate their responses by choosing what best described their child’s behavior on a 4-point Likert scale (1 = not true, 4 = almost always true). Results are then expressed in raw scores and converted to T-scores, so that higher scores mean more social difficulties. In our study, we used two rating forms of the SRS-2: the Preschool Form (age range: 2.5–4.5 years old) and the School-Age Form (age range: 4–18 years old).

To assess adaptive functioning, we used the Vineland Adaptive Behavior Scales, second edition (VABS-II; [42]). This standardized parents interview measures adaptive behaviors from childhood to adulthood in the domains of Communication, Daily-living skills, Socialization, and Motor skills. For each individual domain, a Standardized score (SStd) and a corresponding Adaptive Level, which rank from Low to High levels of adaptation in everyday life, are obtained. The four domain standardized scores are then combined to form the Adaptive Behavior Composite score for individuals aged birth to 6 years 11 months.

In this study, various assessments were used to evaluate cognitive skills depending on the child’s age, language level, symptom severity, and overall ability to attend demanding cognitive tasks. Following others [43], we defined the Best Estimate Intellectual Quotient (IQ) for each participant. When available, we used the Full-Scale IQ (FSIQ) scores assessed with Wechsler Preschool and Primary Scale of Intelligence, fourth edition (*n* = 35). For other children, we calculated a Developmental Quotient (DQ) using the Mullen Scales of Early Learning composite score (MSEL-DQ) (*n* = 55), or Psycho-Educational Profile, third edition, Verbal/Preverbal Cognition scale (PEP-3; VPC-DQ) (*n* = 10).

### 2.3. Eye-Tracking Paradigm

**Figure 1:**
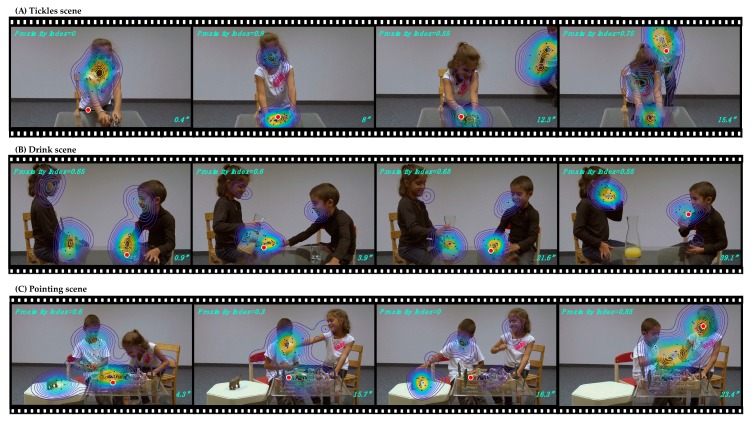
Illustration of the data-driven eye-tracking method. Normative gaze data distribution ("norm"), delimited by contours, was created using gaze coordinates from 36 typically developing children (aged 3.9 ± 0.7). For each TD child, the exact gaze position on a given frame is indicated using blue dots. The three rows show screenshots extracted from the three different social scenes, depicting the norm (colored contours) and gaze data from a 4.5 years old male with ASD (red circle). Proximity values with regards to the norm were obtained on each frame and are indicated in the upper left corner of the given frames. A timestamp for each frame is depicted in the lower right corner.

Gaze data were recorded using a Tobii (https://www.tobiipro.com) TX300 eye-tracking system (300 Hz) with a screen resolution of 1920 × 1200 pixels. The Tobii Studio software (v.3.4.7) was used for the presentation of the stimuli and data collection. Children were seated at approximately 60 cm from the recording screen. After a five-point calibration procedure, children passively watched three custom-designed social scenes that involve a girl and a boy re-enacting everyday situations (Tickles, Drink and Pointing scene, see Figure 1 for an illustration). The Tickles scene (21’’) opens up with the girl playing with animal figurines at the table, until surprised by the boy approaching her from the rear plan, and ends with him tickling her and both of them laughing. The Drink scene (44’’) depicts the two children sharing orange juice; they use non-verbal cues (gestures and facial expressions) to communicate. Finally, the Pointing scene (28’’) depicts the children playing with animal farm figurines and imitating animal sounds; at some point, the girl points towards one distant figurine which the boy subsequently hands over to her, before they continue playing with the figurines. None of the social scenes included verbal communication. The stimuli subtended a visual angle of 45.8° (horizontally) and 26.8° (vertically) and the frame rate was 30 fps. Fixations were extracted using the Tobii IV-T Fixation filter [44] (i.e., Velocity threshold: 30°/s; Velocity window length: 20 ms). Adjacent fixations were merged (maximum time between fixations: 75 ms; the maximum angle between fixations: 0.5°). In this study, only participants who attended each of the three social scenes for more than 50% of the time were included.

To quantify the dynamics of visual behavior in the ASD group without any a priori, we applied a data-driven, custom-developed method [45,46]. This method first defines “normative gaze behavior” (“norm”) using gaze data from the group of TD children, for each frame of the video. This “norm” is then used to quantify how much the gaze pattern of each child with ASD diverges from the typical visual exploration of the social scene. The “norm” is obtained by applying a Gaussian kernel of an adaptive bandwidth [47] at each pair of gaze coordinates and these were linearly summed up to obtain an estimation of the density of gaze data for the TD group (see Figure 1). Obtained density estimation reflects a probability of allocating gaze data at the given location of the visual scene. Subsequently, the gaze allocation for each individual participant with ASD is compared to this norm in a frame-by-frame manner, obtaining a “Proximity index” with values ranging from “0” (gaze coordinates of the child with ASD are situated outside the area attended by the TD group) to “1” (gaze coordinates of the child with ASD are situated in the area that was the most attended by the TD group) (see Figure 1). For each child with ASD, the “Proximity value” is then averaged over the entire duration of the video.

### 2.4. Analyses Strategy

Statistical analyses were conducted using GraphPad Prism v.8, for Mac and Windows (https://www.graphpad.com/scientific-software/prism/). Data-driven measures of visual exploration were obtained using custom software implemented in MATLAB v. 2018b. (https://ch.mathworks.com/products/matlab.html). To describe and characterize the profile of the ASD group in comparison to the TD group regarding the different clinical assessments conducted (ADOS, SSP, SRS-2 Total, VABS-II and Best Estimate IQ), we used Student t-tests and Mann-Whitney tests when measures did not follow a normal distribution according to the D’agostino & Pearson test. Effect sizes were calculated for between-group comparisons of all SSP domains (*η*^2^) and converted into correlation coefficients [48].

Then, within the ASD group, we further examined the relationship between Sensory issues (SSP) and Social difficulties (SRS-2), Adaptive Functioning (VABS-II) on the one hand, and eye-tracking derived measures of social attention (Proximity index) on the other hand, using correlation analyses (Pearson or Spearman depending on the measure distribution following D’agostino & Pearson test). To analyze the Proximity index across the three social scenes selected, we extracted the participants Proximity values for each scene (Tickles, Drink and Pointing). We then calculated the Mean Proximity index across all three scenes for that participant.

Results were considered significant at *p* < 0.05. For the results concerning the four VABS-II subdomains, results were considered significant at the level of *p* < 0.0125 (after applying the Bonferroni correction for multiple comparisons). For the analyses involving the seven modalities of SSP, the results were considered significant at the level of *p* < 0.007. For correlations between all sections of SSP and domains of SRS-2, we used Pearson’s and Spearman’s correlations with a threshold of *p* < 0.0010 (Bonferroni correction for multiple comparisons) (in Supplementary Table S1). The association between all sections of SSP and domains of the VABS-II was also examined using Pearson’s correlations and a threshold of *p* < 0.0018 (Bonferroni Correction) (in Supplementary Table S2).

## 3. Results

### 3.1. Description of The Study Sample

Table 1 describes the clinical characteristics of the ASD and the TD samples. As expected, children with ASD and TD children showed strong significant differences across all used assessments. Our sample of children with ASD showed a moderate to high level of autism symptoms, as illustrated with an average ADOS-CSS of 7.1, whereas all children in the control group had the minimal score of 1 at the ADOS, except one TD child who had a score of 2 (ADOS-CSS; *Mdn_ASD_ = 7, Mdn_TD_* = 1, *U* = 0.500, *p* < 0.001). Compared with the group of TD children, children with ASD also presented more social difficulties as reported by their parents (SRS-2; *Mdn_ASD_ = 62, Mdn_TD_* = 42, *U* = 78.00, *p* < 0.001) and lower adaptive behaviors overall (VABS-II, *M_ASD_* = 81.11, *M_TD_* = 98.52, *t*(*96*) = 6.322, *p* < 0.001). Best Estimate IQ for children with ASD was also lower than TD children (*M_ASD_* = 78.3, *M_TD_* = 113, *t*(*98*) = 7.50, *p* < 0.001) and 39% of children in the ASD group had scores lower than 70.

**Figure 2:**
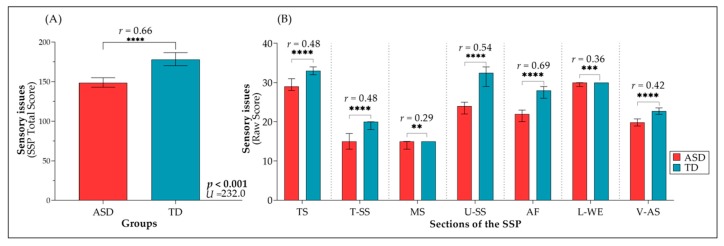
Sensory processing comparison between children with ASD and TD children: (**A**) Total Score of Short Sensory Profile-SSP by groups; (**B**) Raw scores across the seven sections of the SSP by groups.

As illustrated in Figure 2A, Sensory issues were more frequently reported in the group of children with ASD (*Mdn* = 148.5), as compared with the age-matched TD group (*Mdn* = 178.0), *U* = 232.0, *p* < 0.001, *r* = 0.66). Figure 2B depicts between-group differences in all sections of the SSP, revealing that children in the ASD group showed more atypical responses than TD children across all assessed modalities: Tactile Sensitivity (TS; *Mdn_ASD_* = 29, *Mdn_TD_* = 33, *U* = 479, *p* < 0.001, *r* = 0.48), Taste/Smell Sensitivity (T-SS; *Mdn_ASD_* = 15, *Mdn_TD_* = 20, *U* = 507, *p* < 0.001, *r* = 0.48), Movement Sensitivity (MS; *Mdn_ASD_* = 15, *Mdn_TD_*= 15, *U* = 807.5, *p* < 0.004, *r* =0.29), Underesponsiveness/Sensation-Seeking (U-SS; *Mdn_ASD_* = 24, *Mdn_TD_* = 32.5, *U* = 406, *p* < 0.001, *r* = 0.54), Auditory-Filtering (AF; *Mdn_ASD_* = 22, *Mdn_TD_* = 28, *U* = 202, *p* < 0.001, *r* = 0.69), Low Energy/Weak (L-WE; *Mdn_ASD_* = 30, *Mdn_TD_* = 30, *U* = 722, *p* < 0.001, *r* = 0.36), and Visual/Auditory Sensitivity (V-AS; *M_ASD_* = 19.8, *M_TD_* = 22.70, *t*(98) = 4.26, *p* < 0.001, *r* = 0.42). Looking more closely at values of effect size for between-group differences across various domains of sensory functioning, we noted that the difference between the two groups was the most pronounced in sections of Unresponsiveness/Sensation-Seeking (U-SS) and Auditory-Filtering (AF) (Figure 2B).

**Figure 3:**
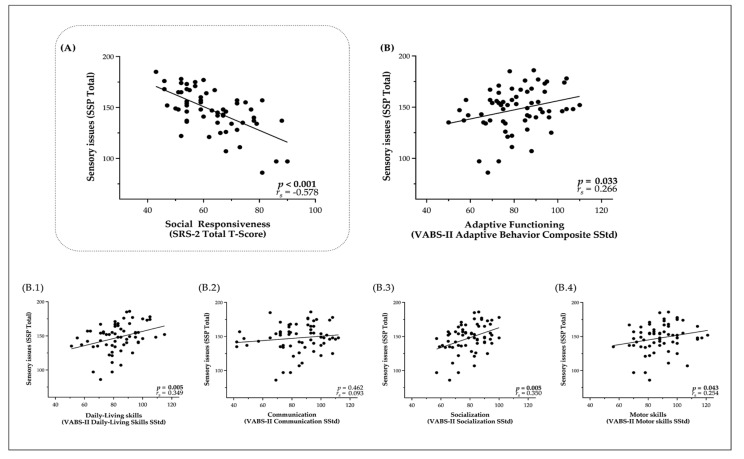
Correlations between Sensory issues (SSP Total score) is the ASD group and: (**A**) Social difficulties (SRS-2 Total T-Score); (**B**) Adaptive Functioning (VABS-II Adaptive Behavior Composite SStd) and subdomains; (**B.1**) Communication (VABS-II Communication SStd); (**B.2**) Daily- living skills (VABS-II Daily-living skills SStd); (**B.3**) Socialization (VABS-II Socialization SStd); and (**B.4**) Motor skills (VABS-II Motor skills SStd).

As shown in Figure 3A, we found a significant negative correlation between SSP Total score and SRS-2 Total T-score in the ASD group (*r_s_* = −0.578, *p* < 0.0001). In other words, parents reporting more frequent and unusual Sensory responses were also reporting more social difficulties in their children. Moreover, analyzing separately the subsections of the SSP, we notice that the strongest correlations with social impairment as measured by the total SRS-2 score originated from the subdomains of Unresponsiveness/Sensory Seeking (U-SS) (*r_s_* = −0.600, *p* < 0.0010) and Auditory-filtering (AF) (*r_s_* = −0.649, *p* < 0.0010) which were significantly correlated with SRS-2 Total Score after the Bonferroni correction for multiple comparisons. More details about the correlation between all subdomains of SRS-2 and SSP questionnaires can be found in Supplementary Materials (Supplementary Table S1). Similar to the ASD group, in the control group, we found a significant negative correlation between SSP Total score and SRS-2 Total T-score (*r* = −0.505, *p* = 0.002). More details about the correlation between subdomains of SRS-2 and SSP Total Score can be found in Supplementary Materials (Supplementary Figure S4). To resume, typically-developing children who have reduced social motivation and present repetitive patterns of behavior (although at subclinical level) also show significantly more sensory issues.

### 3.2. Relationship of Sensory Processing Issues with Social Difficulties and Adaptive Functioning

We also observed that children with ASD who have more sensory issues show more adaptive difficulties in their everyday life (Figure 3A). Indeed, we observed significant correlations between overall Adaptive behaviors (*r_s_* = 0.266, *p* = 0.033), as well as significant correlations between two domains of the VABS-II (Figure 3B.1–2), namely Daily-Living skills (*r_s_* = 0.349, *p* = 0.005) and Socialization (*r_s_* = 0.350, *p* = 0.005). The association between Sensory issues and Motor skills (*r_s_* = 0.254, *p* = 0.043) was no longer significant after Bonferroni correction of the statistical significance threshold (Figure 3B.3). We did not find any significant link between Sensory issues and Communication skills in our sample of children with ASD (*r_s_* = 0.093, *p* = 0.462) (Figure 3B.4).

In addition, we examined the association of all sections of SSP with the different domains of VABS-II (see Supplementary Table S2). After Bonferroni correction for multiple comparisons, we found that the remaining and strongest correlations were seen between Auditory-Filtering and Daily-Living skills (*r_p_* = −0.582, *p* < 0.0018), as well as Socialization (*r_p_* = −0.456, *p* < 0.0018).

### 3.3. Relationship of Sensory Processing Issues with Dynamic Visual Exploration of Social Scenes

**Figure 4:**
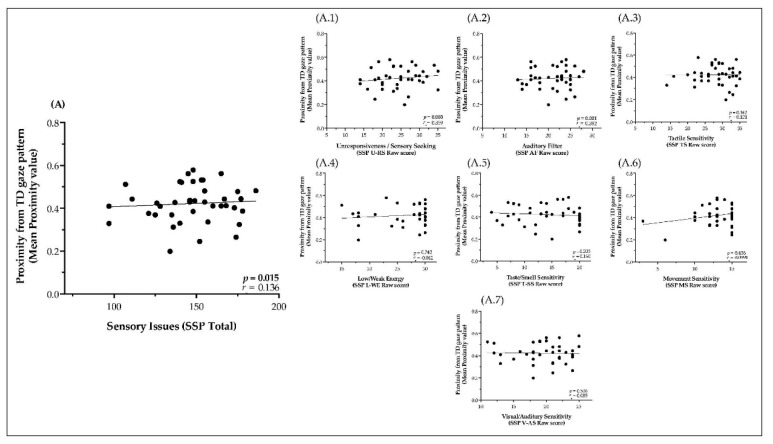
Correlations between Proximity value with: (**A**) Sensory issues (SSP Total score); (**A.1–7**) All seven modalities of the SSP (Raw score) in the ASD group across all three Social Scenes (Tickles, Drink and Pointing scene).

The strong relationship between Sensory issues and parents-reported measures of social difficulties, further inspired us to investigate the link between sensory issues and an unbiased measure of social attention extracted from the eye-tracking. Our custom-developed method (see Methods section for more details) quantifies the dynamic of visual exploration and estimates how much the gaze patterns of each child with ASD diverge from the group of TD children while watching naturalistic social scenes, defining a “Proximity value” for each child in a frame-to-frame manner as illustrated in Figure 1. As depicted in Figure 4A, we found a significant positive correlation between the mean Proximity value for all three social scenes and the scores of the SSP (*r_s_* = 0.303, *p* = 0.015). Precisely, children with ASD who had more Sensory issues showed a more divergent visual exploration of the social scene as compared to the age-matched TD group.

Subsequently, and in order to better understand the processes driving the association between sensory issues and divergence in visual exploration, we correlated the proximity values with scores across the different sections of the SSP (Figure 4A.1–7). After correction for multiple comparisons, we found that children with ASD with more atypical scores in the dimension of Underesponssiveness/Sensation-Seeking (*r_s_* = 0.359, *p* = 0.003) and Auditory-Filtering (*r_s_* = 0.392, *p* = 0.001) were showing more divergent gaze pattern from the one seen in the group of TD children. No significant relationship was found between mean Proximity value and Tactile Sensitivity (*r_s_* = 0.121, *p* = 0.342), Taste/Smell Sensitivity (*r_s_* = 0.160, *p* = 0.205), Movement Sensitivity (*r_s_* = 0.099, *p* = 0.436), Low Energy/Weak (*r_s_* = 0.099, *p* = 0.436), and Visual/Auditory Sensitivity (*r_s_* = 0.085, *p* = 0.506).

Finally, to further untangle the complexity of the divergence in visual pattern between two groups with regards to sensory processing, we conducted analyses on gaze data metrics extracted from the Tobii eye-tracking system. We measured a variety of gaze parameters, namely the mean duration of Fixations (ms), the number of fixations (*n*), the number of saccades (*n*), the first fixation duration (ms), and the first saccade amplitude (°). To identify a potential subgroup of children presenting a more “sticky pattern” of attentional deployment, we categorized the gaze behavior in children with ASD based on the percentage of fixation duration greater than 1000 ms [49]. These analyses did not yield any significant results; more details on the method and results of these analyses can be found in Supplementary analyses (Figure S1, Figure S2, Figure S3).

## 4. Discussion

In this study, we replicated the well-established finding that children with ASD show higher prevalence of sensory processing issues compared with TD children [11]. Examining the different types of sensory-processing issues with the Short Sensory Profile-SSP [34], we found that children with ASD consistently showed more sensory atypicalities compared with TD group across all modalities (Tactile, Taste/Smell, Movement, Underresponsiveness/Sensation-Seeking, Auditory filtering, Low/Weak, Visual/Auditive). However, the strongest group differences were noted in the Underresponsiveness/Sensation-Seeking and Auditory-Filtering sections. Interestingly, these two subsections combine a very large dimension of responses to sensory stimulation, namely Hyporesponsiveness, defined by a lack or diminished response, and Sensation-Seeking [10], covering several domains of functioning. The rather large range of behaviors covered by these subscales might lead to the biggest group differences observed. Furthermore, in the group of children with ASD, the presence of higher levels of sensory issues was related to more pronounced social impairments as reported by parents using the Social Responsiveness Scale (SRS-2). The domains of Underresponsiveness/Sensation-Seeking and Auditory-Filtering were moderately correlated with the levels of social impairment, followed by the domain of Tactile Sensitivity. In this study, we also wanted to go beyond the classical standardized clinical test to measure social impairments and used a custom-developed data-driven eye-tracking method [45,46] to provide a more precise and unbiased measure of visual exploration while watching dynamic social scenes. All children were shown naturalistic social stimuli mimicking frequent social situations (playing with toys, sharing a drink and enjoying a moment of physical play), and our method measured how their gaze patterns diverged from the age-matched group of TD children. The children showing more difficulties of Hyporesponsiveness/Sensation-Seeking type, as well as in the domain of the auditory filtering, were the ones showing the most divergent visual exploration of the social scene. We will discuss below how the general dimension of response to sensory stimulation is crucial to the adequate interpretation of subtle social cues. Finally, we showed that children with ASD presenting more sensory issues had overall poorer adaptive functioning. The adaptive skills in these children were especially compromised in the subdomains of Socialization as well as the Daily Living Skills.

Throughout the entire manuscript, we used stringent criteria to correct for multiple comparisons. The robust results that emerged from the analyses stress the importance of sensory processing for the overall social and adaptive functioning in young children with ASD.

### 4.1. Relation between Sensory Issues and Social Skills

Our results are consistent with previous studies showing the association between sensory processing on the one hand and social impairments on the other. We confirmed that this relationship is already present at a relatively young age, before 6 years old. The association between sensory issues and social impairments showed moderate effect sizes in the ASD group and was mostly driven by higher atypicalities in Underresponsiveness/Sensation-Seeking, Auditor-Filtering and Tactile Sensitivity sections.

Children with ASD who present atypicalities in the domain of Underresponsiveness/Sensation-Seeking either do not notice what is happening around them, or take pleasure in sensations and try to generate additional sensory input (e.g. “Can’t sit still, fidgets”; “Enjoying to make noise for noise’s sake”) [40]. In the ASD group, difficulties of this type were significantly correlated with all subscales of the SRS-2. The strongest association was seen with the Social Motivation domain [41] encompassing difficulties such as initiating or maintaining social interactions (“staring or gazing off into space”), or feeling tense in social situations. As stated, diminished social motivation [50,51,52] is associated with reduced opportunities for social experience, further resulting in social impairment observed in individuals with autism. Previous studies have reported negative correlations between underresponsiveness, language scores and socio-communicative symptoms severity (for a review see [53]). On the other hand, Sensory-Seeking patterns have also been related to a higher prevalence of repetitive behaviors [54]. In return, a higher engagement in over-focused, repetitive predictive patterns of interests, could importantly limit social experiences, thus reducing the opportunities for social learning. Thus, both underresponsive and Sensory-Seeking patterns of interest can have an adverse impact on the social functioning of the individual.

Atypicalities in the section of Auditory-Filtering are described as hypersensibility (“Can’t work if the radio is on”) or hyposensibility (“Does not respond when name is called but you know the child’s hearing is OK”). Difficulties in this domain were moderately related to all subscales of the SRS-2, but most strongly with Communication difficulties, followed by Social Motivation of the SRS-2. Communication difficulties in this scale comprise difficulties understanding and using both verbal and non-verbal means of expression. Difficulties to properly understand communicative cues are crucial to navigating in the social world. The aberrant pattern of sensory response in auditory modality can have deleterious impact on understanding the social interaction and subsequently could impact the willingness (motivation) to do so.

Finally, difficulties in the domain of tactile sensibility concern the discomfort with regards to touch (“Expresses distress during grooming”; “Reacts emotionally or aggressively to touch”) [40]. In the present study, difficulties in this domain were mostly related to diminished scores in Social motivation and Social cognition of the SRS-2 (“Prefer loneliness”, “Difficulties in understanding the subtleties of human interaction”) [41]. In the literature on sensory processing, the difficulties in this domain have been linked to social and emotional distress and were shown to be an obstacle to adequate participation in the social environment [55]. It is plausible to think that hypersensibility to touch would result in diminished motivation to interact with other people, limiting the amount of potentially negative experiences. As described previously, diminished motivation to engage with the social world would substantially hinder the development of social skills [56]. Although our study is not allowing any inference about causal relation between sensory processing and social motivation and cognition, recent prospective studies with infants at risk for ASD [5,12] corroborate such a relationship, showing that the sensory issues predicted reduced social orienting which in turn predicted social impairment at a later age. Sensory issues were also related with autistic traits (measured by SRS-2) in our control group. Our results are in line with previous findings in the general population [19,20], showing that adult individuals presenting higher levels of autistic traits also show more sensory disturbances. This strong relationship between sensory processing issues and autistic traits, evident on the less extreme part of the continuum of autistic traits, once again corroborates the potential implication of sensory processing atypicalities in social functioning of an individual. While there is a lot of work to be done to fully understand the exact nature of the relationship between these two very distinct areas of functioning, our results support the need for further investigation.

We wanted to go a step further in our understanding of the complex interplay between sensory processing on the one hand and processing of social information. To precisely quantify visual exploration, we obtained unbiased eye-tracking-derived measures using naturalistic social scenes. These dynamic measures of visual behavior inform us frame-by-frame about the relative divergence (or Proximity) of the gaze position of each child with ASD with regards to the “norm” derived from cumulative gaze distribution from TD group for that specific frame [45,46]. Our results indicate that the difficulties in Underresponsiveness/Sensation-Seeking and Auditory-Filtering were strongly reflected onto the gazing pattern of children with ASD. Children with ASD having more difficulties in Underresponsiveness/Sensation-Seeking and Auditory-Filtering domains also showed a pattern of visual exploration that was radically different from the one seen in TD group. To the best of our knowledge, this study is the first to relate the complexity of the visual exploration pattern of dynamic naturalistic social scenes with sensory-processing atypicalities in children with ASD. The stimuli used in the current study were ecological and depicted various situations from everyday life. Our aim was to create a type of stimuli that would probe for the visual exploration that was the most representative of the usual functioning of the individual. As stated earlier, sensory atypicalities can significantly impact the processing of social information [21], and our eye-tracking results provide more support for this relationship.

In further analyses of the eye-tracking data detailed in the Supplementary Materials, we aimed at a more thorough understanding of the processes that were generating the divergence in the gaze pattern in the ASD group. Our hypothesis was that the pattern of visual exploration in the ASD group might be less dynamic, with slower shifts between various elements of the scenes and characterized by “sticky attention” to particular details of the scene. Slower attention disengagement has been shown to characterize the visual pattern in Sibs ASD who will develop ASD already at 6 months of life [57] and is known to persist throughout childhood [58]. Our results, however, did not corroborate this hypothesis (for more details please refer to Supplementary Materials (Figure S1, Figure S2, Figure S3). We indeed found no significant between-groups differences concerning the overall fixation duration or the number of fixation/saccades (as both groups were selected based on the good screen attendance; a lesser number but of larger saccades would be indicative of a “sticky attention “pattern). While not reaching a significant threshold, we noticed that the ASD group children compared with the TD group had longer fixations (longer than 1s) which we classified as “staring” in concordance with previous findings from studies of naturalistic deployment of visual attention [49].

The social scenes used in this study did not include any language, which is an important “top-down” moderator of gaze behavior. Thus, the next step would be to measure the relative contribution of more basic “bottom-up” attentional processes to the divergence in visual pattern that we detected. These basic, “bottom-up” processes are shown to contribute importantly to the allocation of visual attention in adults with ASD while watching static images [59]. Visual attention of adult participants with autism is shown to be more attracted to the image center and towards low-level then higher (semantic)-level saliency elements, like faces or objects of another’s gaze. These attentional atypicalities seem to be more evident at later processing stages where the semantic-level influence is generally the most dominant [59]. When actually attending to social information, for example, while taking photographs of people, the attention of adults with ASD seems to be attracted to unusual aspects of the social environment, such as less-expressive faces and a tendency toward taking a tilted perspective [60]. However, these atypicalities are a product of a lifetime of experience and it is highly important to understand how they emerge in the development and what is the relative share of bottom-up and top-down processes in orchestrating visual attention deployment in the younger population while watching dynamic complex social stimuli.

### 4.2. Relationship Between Sensory Issues and Adaptive Functioning

In addition to the influence of social functioning, early sensory-processing atypicalities are known to significantly impact the overall level of adaptive functioning in individuals with ASD [31]. Adaptive functioning refers to a set of conceptual, practical and social skills performed in the everyday life [61]. Adaptive skills levels are reported to be more predictive of the outcome in individuals with ASD than the general intellectual functioning [62]. Adaptive skills are generally shown to be lower in ASD than in other neurodevelopmental conditions [63] and this is even for individuals on the higher functioning end of the spectrum [17]. It is thus of great importance to understand the hindering elements of a good functional outcome. Recent studies quite consistently reported a link between the sensory-processing atypicalities and adaptive functioning [29,30]. A recent longitudinal study with children aged 2–12 years old showed that the relative contribution of sensory issues to overall adaptive functioning varied across different subtypes of sensory difficulties, with increased Hyporesponsiveness and Sensory-Seeking behaviors being related to a poorer adaptive outcome a year later [31]. In a recent study using a longitudinal design [29], the authors showed that Hyporesponsiveness measured earlier around 3–8 years old predicted poorer adaptive skills 4 years later. Another large study with children aged from 2–17 years old corroborated findings of increased sensory-seeking behaviors, which were negatively related with overall adaptive functioning [30].

In the current study, we observed that the overall level of sensory difficulties was negatively correlated with the adaptive functioning in preschoolers with ASD. The most compromised subdomains of adaptive functioning were Daily Living Skills and Socialization. Previous studies have reported the negative association between sensory issues and autonomy in daily activities [64,65], highlighting the importance of addressing the difficulties in this domain for a better quality of life in these individuals. In our study, the strongest association of sensory difficulties with adaptive functioning was detected for the Auditory-Filtering subsection of the SSP [34]. Auditory-Filtering atypicalities negatively correlated with Socialization and daily Living skills subdomains of Vineland. An alteration in the processing of the auditory information may have important consequences on the adaptation and autonomy and needs to be addressed and treated as early as possible in view of the importance of sensory processing in the developmental cascade.

## 5. Conclusions

Our study identified many areas of interactions between sensory issues and social deficits in preschoolers with ASD, and provided a first account of strong links between sensory difficulties and eye-tracking measures of social cognition. However, our study bears several limitations. First, we used a cross-sectional design with correlational analyses, which limits the scope of possible causal inferences. Second, we only focused on children older than 3 years old, as that the SSP uses different versions for children younger and older than 3 years old, limiting possibilities to use a sample of participants with a broader age range. Given the developmental nature of autism symptoms, it would be interesting to focus on sensory processes already in the first year of life, to better delineate their contribution to the future functioning outcome of the child. Third, here we used the shorter form of the Sensory Profile [40], as an indirect measure of sensory-processing difficulties. While the short version of the Sensory Profile used in this study still allows a very valid and pertinent description of the sensory difficulties, using the longer form of the Sensory Profile [40] or other more direct measures of sensory processing [53] would have resulted in a more fine-grained characterization of sensory difficulties (hypo- vs. hyper- responsivity across different domains). Despite these limitations, the strength of the association between sensory issues and higher-order constructs such as social deficits and adaptive functioning observed in the current study paves the way for future longitudinal studies to better understand the emergence of autism symptoms.

## Figures and Tables

**Table 1:** Description of the ASD and TD samples.

Measures	ASD (*n* = 68)	TD (*n* = 36)	
	*Mean* (*SD*)	*Mean* (*SD*)	*p value* *
Age	4.0 (0.8)	3.9 (0.7)	*p* = 0.769^2^
Best Estimate IQ	**78.3 (26.2)**	**113 (13.4)**	***p* < 0.001^1^**
ADOS-CSS	**7.1 (1.7)**	**1.0 (0.2)**	***p* < 0.001^2^**
SSP Total	**147.7 (20.5)**	**176.6 (11.5)**	***p* < 0.001^2^**
SRS-2 Total	**63.7 (11.6)**	**43.2 (5.2)**	***p* < 0.001^2^**
VABS-II Adaptive Behavior Composite	**81.0 (13.4)**	**98.5 (11.9)**	***p* < 0.001^1^**

Note. * *p value* of parametric *Student t-tests^1^* and nonparametric *Mann-Whitney tests^2^* of differences between the ASD and TD group. Significant results are shown in bold.

## Supplementary Materials

The following are available online at https://www.mdpi.com/2077-0383/8/10/1508/s1, Table S1: Correlations between SRS-2 domains and sections of the SSP in children with ASD (n = 64), Table S2: Correlations between VABS-II domains and sections of the SSP in children with ASD (n = 64), Figure S1: Correlations between Sensory issues (SSP Total score) in the ASD group and gaze data in all three Social scenes: (A) Mean Fixations Duration, (B) Number of fixations, (C) Number of Saccades, (D) First Fixation duration, and (E) First Saccade amplitude, Figure S2: (A.1–2) Correlations between Sensory issues (SSP Total score), and Proximity to TD gaze pattern (Proximity mean) with the percentage of Fixations duration greater than 1000ms in the ASD group; (B.1–2) Sensory processing (SSP Total score), and Proximity to TD gaze pattern (Proximity mean) comparisons between children with ASD who had fixations greater than 1000ms (Longer fixations) and fixations lesser than 1000ms (Shorter fixations); (C) Percentage of fixations greater than 1000ms comparison between ASD and TD groups. Figure S3: (A.1–2) Correlations between Sensory issues (SSP Total score), and Proximity to TD gaze pattern (Proximity mean) with the percentage of Fixations duration greater than 1000ms in the ASD group; (B.1–2) Sensory processing (SSP Total score), and Proximity to TD gaze pattern (Proximity mean) comparisons between children with ASD who had fixations greater than 1000ms (Longer fixations) and fixations lesser than 1000ms (Shorter fixations); (C) Percentage of fixations greater than 1000ms comparison between ASD and TD groups. Figure S4. Correlations between Sensory issues (SSP Total score) and Social responsiveness scale (SRS-2) in a group of typically developing children.
