# Supplementary material for: Sensory Processing Issues and Their Association with Social Difficulties in Children with Autism Spectrum Disorders
Source: J Clin Med. 2019 Sep 20;8(10):1508. doi: 10.3390/jcm8101508 (PMC6833094; doi:10.3390/jcm8101508)
Supplement: Supplementary file 1 [file jcm-08-01508-s001.zip › Supplementary analyses.pdf]

Supplementary analyses

To better understand whether the visual exploration of social scenes was related with more basic gaze behaviors such as longer/shorter fixations or altered saccade dynamics, we extracted various gaze metrics for each scene: the mean duration of Fixations (ms), the number of fixations (n), the number of saccades (n), the first fixation duration (ms) and the first saccade amplitude (°).

We then explored whether these basic metrics (mean, number, duration of fixations and amplitude of saccades) were related with measures of sensory processing, using correlation analyses (Pearson and Spearman depending on measure distribution). We then compared these gaze metrics between the ASD and TD groups (Student t-tests and Mann-Whitney according to measures distribution). We later categorized children with ASD based on the percentage of fixations duration greater than 1000 ms (Longer fixations vs. Shorter fixations) on the one hand, and according to saccades amplitude greater than 20° (Bigger saccades vs. Smaller saccades) on the other hand. We conducted comparison analyses between the groups created for each parameter (Student t-tests and Mann-Whitney according to measures distribution). For all analyses, results were considered significant at $p < 0.05$.

1. Association of Sensory Processing and Gaze Data Metrics

Within the group of children with ASD, we did not find any significant relationship between sensory processing issues and mean Fixations duration (*Figure S1A*), nor with the number of Fixations children with ASD made on average across all the social scenes (*Figure S1B).* We also did not observe any significant relationship between Sensory processing and the Number of Saccades (*Figure S1C*), First Saccade amplitude (*Figure S1D*), nor First Fixation duration ($r_s = 0.144$, $p = 0.288$) (*Figure S1E).*

2. Sensory Processing and Mean Proximity for Fixations Duration Greater than 1000 ms

We further explored whether patterns of "sticky attention", as defined with fixations greater than 1000 ms were related with sensory issues in the group of children with ASD. As presented in Figure S2A1-2, there was a tendency for more sensory processing difficulties in children who showed larger percentage of fixations greater than 1000ms, but this did not reach significance level ($r_p = 0.538$, $p = 0.079$). We however did not find any relationship between the percentage of fixations longer than 1000ms and how children with ASD visually explored social scenes when compared to TD gaze pattern ($r_p = -0.130$, $p = 0.305$).

To further examine the non-significant tendency for a relationship between sensory issues and larger percentage of sticky fixation, we further subdivided the group of children with ASD into two subgroups, using a cutoff at the median (3.74%) . Even in these further analyses (Figure S2B1-2), children who presented a higher percentage of Longer fixations did not differ from children who had Shorter fixations regarding Sensory processing ($t(62) = 1.015$, $p = 0.314$) or how far their gaze pattern diverged from that of TD children ($U = 432.5$, $p = 0.289$). We finally found no difference between ASD and TD groups according to the percentage of fixations greater than 1000 ms ($U = 1059$, $p = 0.507$) (*Figure S2C).*

3. Sensory Processing and Mean Proximity for Saccades Amplitude Greater than 20°

We investigated aspects of global visual exploration using saccades amplitude; children making saccade focused on a particular part of the screen might have a different way of processing percepts of their environment and social information than children who made bigger saccades across the screen.

In our sample, the prevalence of presenting saccade amplitudes greater than 20° was not related to Sensory issues ($r_s = 0.189$, $p = 0.136$) or Proximity to TD gaze pattern ($r_s = 0.189$, $p = 0.136$) in children with ASD (*Figure S3A1-2*).

We also created two subgroups of children with ASD according to the percentage of saccades greater than 20°, using a cutoff at the median (0.21%). Children with ASD who tended to present

bigger saccades amplitudes (>20°) differed in their overall Sensory processing when compared with children with ASD who tended to make smaller saccades amplitude (<20°) (U = 317.0, p = 0.008). This difference between the two subgroups of children with ASD was however not significant with regards to Proximity to TD gaze pattern (U = 554.5, p = 0.662) (*Figure S3B1-2*).

Figure S3C shows that children with ASD and TD children presented no significant difference in the percentage of saccades with an amplitude greater than 20° while they explored social scenes (U = 1076, p = 0.559).

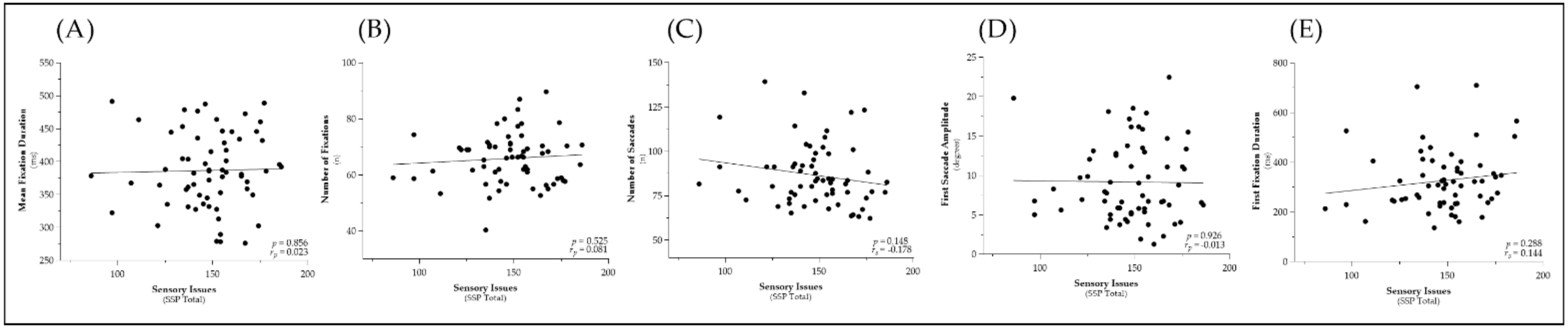

Figure S1. Correlations between Sensory issues (SSP Total score) in the ASD group and gaze data in all three Social scenes: A) Mean Fixations Duration, B) Number of fixations, C) Number of Saccades, D) First Fixation duration, and E) First Saccade amplitude.

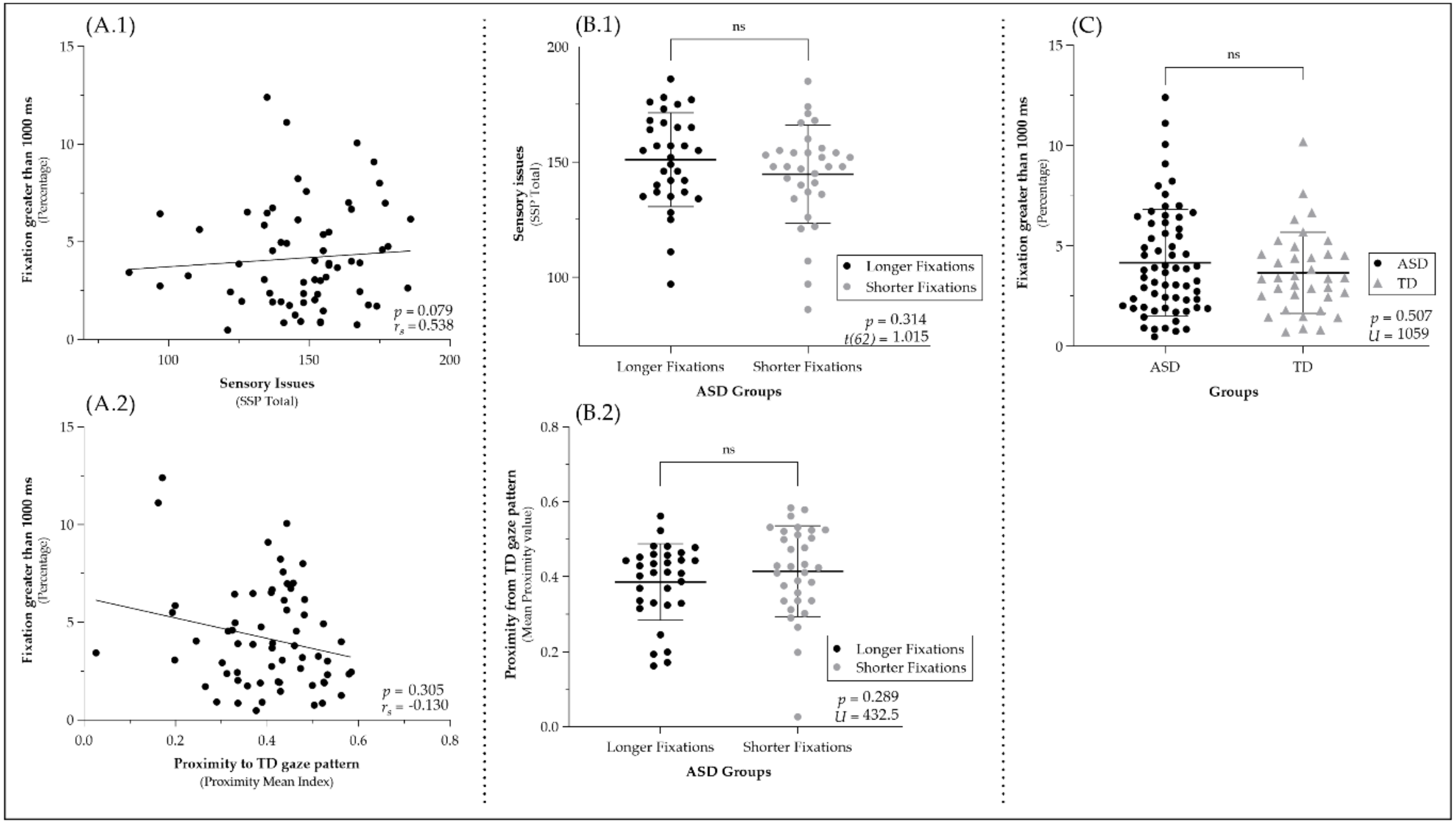

Figure S2. A.1-2) Correlations between Sensory issues (SSP Total score), and Proximity to TD gaze pattern (Proximity mean) with the percentage of Fixations duration greater than 1000ms in the ASD group; B.1-2) Sensory processing (SSP Total score), and Proximity to TD gaze pattern (Proximity mean) comparisons between children with ASD who had fixations greater than 1000ms (Longer fixations) and fixations lesser than 1000ms (Shorter fixations); C) Percentage of fixations greater than 1000ms comparison between ASD and TD groups.

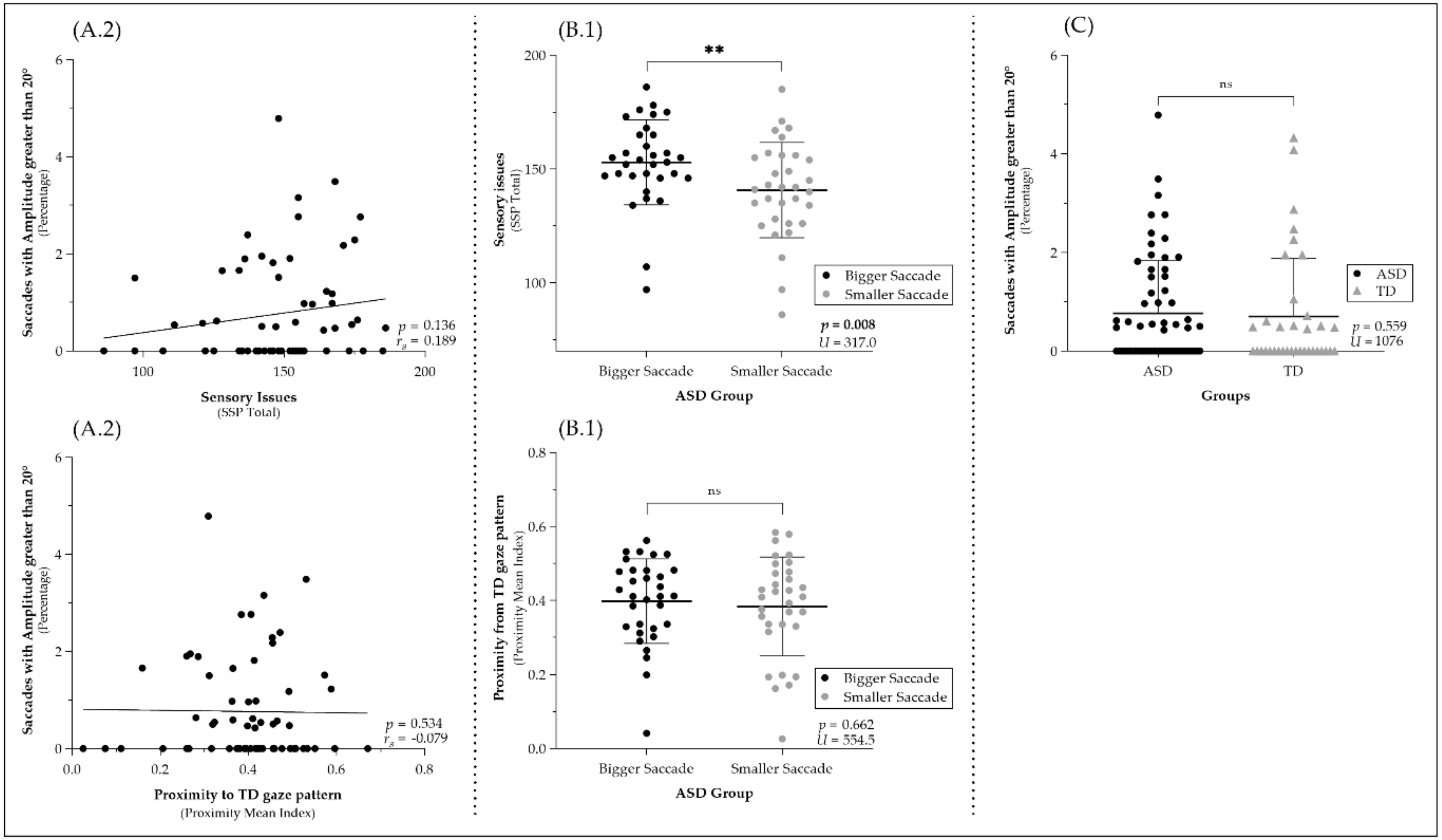

Figure S3. A.1-2) Correlations between Sensory issues (SSP Total score) and Proximity to TD gaze pattern (Proximity mean) with the percentage of Saccades amplitude greater than 20° in the ASD group; B.1-2) Sensory processing (SSP Total score) and Proximity to TD gaze pattern (Proximity mean) comparisons between children with ASD who had saccades greater than 20° (Bigger saccades) and saccades lesser than 20° (Smaller saccades); B) Percentage of Saccades amplitude greater than 20° comparison between ASD and TD groups.
