# Supplementary material for: Sensory Processing Issues and Their Association with Social Difficulties in Children with Autism Spectrum Disorders
Source: J Clin Med. 2019 Sep 20;8(10):1508. doi: 10.3390/jcm8101508 (PMC6833094; doi:10.3390/jcm8101508)
Supplement: Supplementary file 1 [file jcm-08-01508-s001.zip › Supplementary TableS1.pdf]

Table S1. *Correlations between SRS-2 domains and sections of the SSP in children with ASD (n = 64)*

Measures		***SRS-2 Domains (T-score)***							
		Total	Communication	Cognition	Awareness	Motivation	RRB	SCI	RRBI
SSP Sections (Raw score)	Total	**-.578****	**-.518****	**-.513****	-.282*	**-.542****	**-.566****	**-.529****	**-.620****
	Tactile[1]	**-.465****	**-.429****	**-.423****	-.264*	**-.484****	-.373**	**-.440****	**-.455****
	Smell/Taste[1]	-.290*	-.272*	-.246	.007	-.287*	-.353**	-.264*	-.367**
	Movement[1]	-0,249	-.211	-.236	.004	-.318*	-.279*	.096	-.319*
	Unresponsiveness/Seeking[2]	**-.600****	**-.492****	**-.513****	**-.431****	**-.548****	**-.500****	**-.547****	**-.691****
	Auditory Filter[1]	**-.649****	**-.601****	**-.582****	**-.439****	**-.585****	**-.472****	**-.638****	**-.525****
	Low/Weak[1]	-0,192	-.207	-.193	-.087	-.146	-.264*	-.162	-.204
	Visual/Auditory[2]	-.299*	-.247	-.352**	-.068	-.256	-.308*	-.301*	-.265*

Note. *Spearman*[1] et *Pearson*[2] correlations were conducted depending of measures distribution, following D'agostino & Pearson test. Significant results after Bonferroni correction are shown in bold (p < 0.0010).

** Correlation is significant at the 0.01 level (2-tailed).

* Correlation is significant at the 0.05 level (2-tailed).
