# Supplementary material for: Sensory Processing Issues and Their Association with Social Difficulties in Children with Autism Spectrum Disorders
Source: J Clin Med. 2019 Sep 20;8(10):1508. doi: 10.3390/jcm8101508 (PMC6833094; doi:10.3390/jcm8101508)
Supplement: Supplementary file 1 [file jcm-08-01508-s001.zip › Supplementary TableS2.pdf]

Table S2. *Correlations between VABS-II domains and sections of the SSP in children with ASD (n = 64)*

Measures		*VABS-II Domains (SStd)*				
		Total	Communication	Daily-Living skills	Socialization	Motor skills
SSP Sections (Raw score)	Total	-.283*	-.133	-.513**	-.385**	-.220
	Tactile[1]	-.237	-.097	-.423**	-.313*	-.210
	Smell/Taste[1]	-.083	-.006	-0,246	0,209	-.019
	Movement[1]	-.030	-.172	-0,236	0,074	-.016
	Unresponsiveness/Seeking1	-.223	-.150	-.513**	-.276*	-.159
	Auditory Filter[1]	-.364**	-.241	**-.582****	**-.456****	-.263*
	Low/Weak[1]	-0,197	-.102	-0,193	-.229	-.229
	Visual/Auditory1	-.201	-.092	-.352**	-.214	-.166

Note. *Pearson*[1] correlations were conducted depending of measures distribution, following D'agostino & Pearson test. Significant results after Bonferroni correction are shown in bold ($p < 0.0018$).

** Correlation is significant at the 0.01 level (2-tailed).

* Correlation is significant at the 0.05 level (2-tailed)
