# Supplementary figures and images for: Sensory Processing Issues and Their Association with Social Difficulties in Children with Autism Spectrum Disorders
Source: J Clin Med. 2019 Sep 20;8(10):1508. doi: 10.3390/jcm8101508 (PMC6833094; doi:10.3390/jcm8101508)

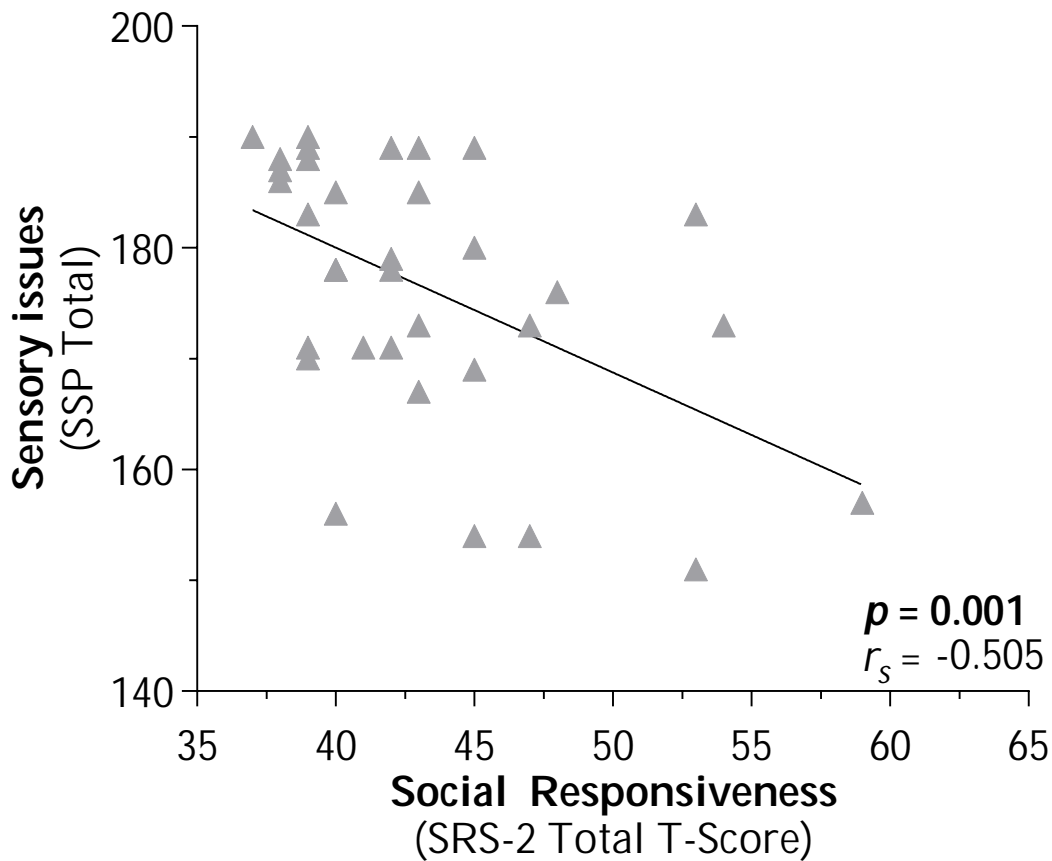

Supplement: Supplementary file 1 [file jcm-08-01508-s001.zip › Figure_S4.pdf]
